# Welcome to the lab

**DOI:** 10.7554/eLife.79627

**Published:** 2022-05-03

**Authors:** Andrey Andreev, Valerie Komatsu, Paula Almiron, Kasey Rose, Alexandria Hughes, Maurice Y Lee

**Affiliations:** 1 Division of Biology and Biological Engineering, California Institute of Technology Pasadena United States; 2 University of Southern California Los Angeles United States; 3 EIT Food Northwest Reading United Kingdom; 4 Zilkha Neurogenetic Institute, Keck School of Medicine, University of Southern California Los Angeles United States; 5 Westat Rockville United States; 6 Genome Institute of Singapore Singapore Singapore

**Keywords:** research culture, onboarding, early-career researchers

## Abstract

Having a formal onboarding procedure for new lab members can lead to a happier and more productive working environment.

There is a lot to learn when starting work in a new research group or laboratory – from everyday laboratory logistics to specialized scientific techniques. Making new hires feel comfortable and getting them working efficiently benefits everyone involved. In the non-academic world, acquainting new employees with company policies and equipment is known as onboarding, and is widely used in industry to improve job performance and satisfaction.

A crucial part of this process is the ‘onboarding package’ given to new staff. This package typically outlines the company’s culture and expectations, explaining tasks such as scheduling and using annual leave. However, most academic labs do not have a formal process for introducing new members to their group, which can sometimes result in mismatched expectations and unnecessary confusion that leads to inefficiency.

This article discusses the documents and information that should be included in an onboarding package. By implementing a program tailored to their lab, group leaders and principal investigators (hereafter referred to as PIs) can integrate new members (which can include graduate students, undergraduates, and staff) more effectively, allowing employees to hit the ground running as soon as they join the lab (Goldstein and Avasthi, 2021).

## ‘First-day’ checklist

Providing new members with a list of essential items they should receive or have access to can accelerate adjustment to the lab (see Box 1 for an example). Such a checklist may include the following:

- How to access the physical lab space (i.e., obtaining keycard access or keys)
- Information about lab-related rooms (such as the cell culture room or vivarium), core facilities, and shared equipment
- Link(s) to university-provided email services
- Contact information for department administrators and facility managers
- Email listservs and Slack channels
- Emergency phone numbers (lab manager, PI, senior graduate student, etc.)
- Links to transportation options/services
- Locations of university-designated community rooms or kitchens
- Department payroll information and pertinent links
- A dedicated computer along with lab-provided office supplies and electronics

Box 1. **Examples of onboarding documents.**

Here we provide a list of example documents that can be used to help build an onboarding package for new lab members. All resources are shared with permission.

1. A ‘First-day’ checklist outlining the essential items a new employee should receive and have access to when they arrive in the lab (compiled by the authors). Readers are invited to use and share this template. Link: https://docs.google.com/document/d/1LS8DL-aUL4L2BZeUcg89XYMnPFTzu034JiOmeTGewK4/edit.
2. A timeline and milestone document provided by the Sammons Lab (State University of New York at Albany) listing the key events PhD students are expected to complete each year. Link: http://thesammonslab.org/labmanual/grad_student_info/.
3. An example of an ‘Individual Development Plan’ for graduate students provided by the Bioscience Department at Stanford University. Link: https://biosciences.stanford.edu/current-students/idp/.
4. A lab philosophy shared over Twitter by Dr. Iain Cheeseman (Massachusetts Institute of Technology), outlining his general approach to science: https://twitter.com/iaincheeseman/status/1432654418437095431.
5. A detailed lab philosophy document created by Dr. Moin Syed (University of Minnesota) outlining how he manages graduate students and certain aspects of the research process, such as funding and publishing. Link: https://docs.google.com/document/d/1wa067HF3iBv5M0_ao6M1GzPg4mUof_PoVlEAKyIn8Xk. Originally shared in a Twitter thread: https://twitter.com/syeducation/status/1025117254239891463.
6. Focused combination of lab policies, philosophy, and best practices published on lab website by Dr. Prachee Avasthi. Link: http://www.avasthilab.org/lab-policies-and-tips/.

While we have provided a basic checklist for new lab members, it is important to note that universities or individual departments may also offer ‘orientation checklists’ for new employees and students. It can be beneficial to supplement the onboarding package with material provided by the university; however, we suggest not relying on this information entirely as it will not be specific to the lab environment and may lack important details.

## A general timeline

We recommend including the typical milestones and timelines expected for incoming lab members within the onboarding package; this can help ensure that the expectations of the new lab member are aligned with those of the PIs. Timelines should include the frequency of ‘check-in’ meetings between the PI and the new lab member. These meetings are crucial to define and evaluate an employee’s short- and long-term goals, improving workflow optimization.

Agreed-upon timelines are particularly useful for PhD students who must achieve specific milestones before the university awards their diploma. PhD timelines can include department-specific requirements, such as coursework and teaching responsibilities, and goals specific to the research project (see example in Box 1). To increase productivity, PIs can construct a similar timeline for other lab members, such as postdocs and technicians. However, the milestones included in the timeline will need to be tailored to each individual based on their project, lab role, and contract length.

## A lab philosophy

PIs often have different views on certain aspects of the scientific process, such as publishing, collaborations, and conferences. However, these attitudes tend to be assumed and not formally stated. Communicating the lab’s scientific policies and core values as part of the onboarding package can help new (and existing) members be more aware of the PI’s expectations. Below are some pragmatic questions to consider when putting this ‘lab philosophy’ document together:

- Does the lab post papers as preprints?
- How is authorship and author order decided when submitting papers?
- How often are lab members expected to travel to conferences?
- How is travel funded, and what type of work can be presented?
- What value does the PI put on academic service (Armani et al., 2021), such as organizing or volunteering within or outside the university?
- Are postdocs and PhD students encouraged to apply for grants?
- Does the lab make data and code openly available?

These questions provide a framework for what might be useful to include in a lab philosophy document. Other examples of lab philosophies can be seen in Box 1.

## Working hours

Striking a proper work-life balance can be challenging for many in academic research labs. Surveys of PhD students (pre- and post-pandemic) have found that approximately 40% were dissatisfied with their work-life balance, 76% worked more than 40 hours per week, and one in three students were at risk of developing a mental health disorder (Woolston, 2019; Levecque et al., 2017; Forrester, 2021). Postdocs, faculty, and staff face similar challenges (Woolston, 2020). Indeed, 30% of faculty reported regularly feeling exhausted in a 2020 survey (Businesswire, 2021), and technicians, such as research vivarium personnel, also reported suffering from workplace-related stress due to long hours (Murray et al., 2020).

Providing realistic expectations regarding working hours and vacation time in the onboarding document can help mitigate burnout-inducing scenarios. This can include: when and for how long employees are expected to physically be in the lab; how much vacation time employees are entitled to; how far in advance employees should notify the PI before taking time off; the PI’s stance on taking mental health days.

Starting these conversations early – when someone first joins the lab – can help alleviate some of the stress of balancing work and personal life.

## Division of labor

Most labs do not have dedicated staff responsible for making stock solutions, managing laboratory animals or cell cultures, and keeping equipment in working condition. Clearly outlining the division of labor for these kinds of tasks can help the lab run more smoothly and equitably.

Women are more likely than men to assume or be delegated to support roles regardless of their job description (Dishman, 2015). Eliminating ambiguity surrounding general lab maintenance will help minimize burdens that can take away from research productivity, which disproportionately impacts women and people of color. PIs can also make lab managers and technicians responsible for supervising lab tasks, empowering them to enforce obligations that other members of the group have agreed to take on.

## Conclusion

A single onboarding package is unlikely to comprehensively address every new employee’s unique needs and situations. However, many tasks, practices, and tools are common to research environments. It is valuable to construct a basic onboarding template that can be expanded to include job-specific considerations to help new members integrate into the lab smoothly and quickly.

An onboarding package is a living document that requires time and effort from the PI and all lab members to remain relevant. We hope to streamline this by outlining topics of consideration, and providing resources to help with the production and maintainance of these documents. We are confident that the energy invested in constructing detailed onboarding practices will pay off in increased productivity and job satisfaction in academic research.
